# Consumer Behavior in the Choice of Mode of Transport: A Case Study in the Toledo-Madrid Corridor

**DOI:** 10.3389/fpsyg.2017.01011

**Published:** 2017-06-20

**Authors:** Ana I. Muro-Rodríguez, Israel R. Perez-Jiménez, Santiago Gutiérrez-Broncano

**Affiliations:** ^1^Econometrics Area, Department of Spanish and International Economy, Econometrics, History and Economic Institutions, University of Castilla-La Mancha, Cobertizo San Pedro MártirToledo, Spain; ^2^Business Administration Department, University of Castilla-La Mancha, Talavera de la ReinaToledo, Spain

**Keywords:** consumer behavior, choice of service, transportation, modeling, discrete choice, logit, willingness to pay

## Abstract

Within the context of the consumption of goods or services the decisions made by individuals involve the choice between a set of discrete alternatives, such as the choice of mode of transport. The methodology for analyzing the consumer behavior are the models of discrete choice based on the Theory of Random Utility. These models are based on the definition of preferences through a utility function that is maximized. These models also denominated of disaggregated demand derived from the decision of a set of individuals, who are formalized by the application of probabilistic models. The objective of this study is to determine the behavior of the consumer in the choice of a service, namely of transport services and in a short-distance corridor, such as Toledo-Madrid. The Toledo-Madrid corridor is characterized by being short distance, with high speed train available within the choice options to get the airport, along with the bus and the car. And where offers of HST and aircraft services can be proposed as complementary modes. By applying disaggregated transport models with revealed preference survey data and declared preferences, one can determine the most important variables involved in the choice and determine the arrangements for payment of individuals. These payment provisions may condition the use of certain transport policies to promote the use of efficient transportation.

## Introduction

Transport is one of the most important services of a developed society. The growing need for mobility of people motivated mainly by the spatial differences of the locations to which people need access to makes transport modeling a matter of spatial importance for any developed country. One of the main problems to be analyzed is how individuals move or what their mobility patterns are. This issue is fundamental to the proper planning of the transport system. An efficient transport system must serve the mobility needs of individuals, for this it must use the necessary tools to be able to plan this mobility.

The individual faces, decisions daily between different alternatives of choice, whether goods or services, conditioned by the qualities or attributes of the different options available (McFadden, 2001b; Ye et al., 2007; Chowdhury and Ceder, 2016; Houdek, 2016). In order to determine the variables that affect their choice and to determine the probability of choosing between different available options, disaggregated demand models are used (McFadden, 1981; Schakenbos et al., 2016).

Disaggregated demand models are also called discrete choice models, due to the characteristics variable of choice that can be the mode of transport; this variable is discrete, due to the qualitative nature of the individual's response and also a finite number of responses are shown (Ben-Akiva and Lerman, 1985; Train, 2003; Navarette and Ortuzar, 2013). These models have the ability to predict individual decisions and joint decisions and thus serve as a basis for policy planning (St-Louis et al., 2014).

In order to try to predict the behavior of users regarding modal choice, we will focus on the disaggregated demand models based on the Theory of Choice, in which the traveler maximizes its utility (McFadden, 2001a; Train, 2003). These disaggregated demand models are based on the theory of discrete choice, to determine the probability of choosing the different alternatives which the individual counts with (Martín et al., 2011).

Most of the models used for travel behavior applications are based on utility theory (McFadden, 1974; Domencich and McFadden, 1975; Manski, 1977; Williams, 1977; de Dios Ortúza and Willumsen, 2001), which assumes that the preference of choice of an alternative is captured by a value, called utility, and decision-making selects the alternative in the set of choices most satisfactory (Taniguchi et al., 2014).

This concept, used by the microeconomic theory of the consumer, presents strong limitations for practical applications, since the complexity of human behavior suggests that the decision rule must include a probabilistic dimension (Simon, 1957; Sandoval, 1994; McFadden, 2001b).

Some models assume that the decision rule is intrinsically probabilistic, and even a complete understanding of the problem does not surpass uncertainty (Thurstone, 1927; Quandt, 1956; Luce, 1959; Tversky, 1969; McFadden, 1981). Others consider that the decision rules of individuals are deterministic and motivate uncertainty to the analyst's limited ability to observe and grasp all the dimensions of the election process because of its complexity (Aguado Franco, 2012).

A fundamental assumption in the choice process is that decision making is assumed to have “rational behavior” and implies that a decision maker is a “maximize” of utility and, in the same circumstances, will repeat the same choice, in addition to the transitivity of preferences (Domencich and McFadden, 1975; McFadden, 2001b; de Dios Ortúza and Willumsen, 2001; Train, 2003).

For the estimation of disaggregated demand models, survey data are required. Two types of surveys, revealed preferences, and declared preference surveys can be differentiated. The revealed preference surveys, which try to reflect the current behavior of individuals in their travel decisions and the declared preference surveys, provide us with data that try to reflect how travelers share a certain hypothetical situation (Espino, 2003).

The Toledo-Madrid corridor has HST, bus and car, as alternative modes of transport. In addition, the completion of the Atocha-Chamartín-Airport railway corridor will allow the connection between the Spanish high-speed network and Madrid's international airport. This will favor the cooperation of operators to offer better transport services to travelers (Muro, 2012): new figures of transport tickets, shorter connection times, integrated baggage management, information visualization, etc. (Eurocontrol, 2005b).

In this context, the research work on the “Toledo case” analyzes the current state of mobility in the Toledo-Madrid corridor and, on the other hand, changes the situation in a scenario of new transport infrastructures. This makes it possible to formulate policies at the level of passenger mobility to mitigate the most harmful effects of transport, such as congestion, environmental damage (INFRAS IWW, 1995, 2000, 2004; Intergovernmental Panel on Climate Change (IPCC), 1999; Aviation Environment Federation (AEF), 2000; Whitelegg and Williams, 2001; IATA, 2003; Steer Davies Gleave, 2006; CE Delft, 2008, 2011), by promoting complementarity between modes (Watkiss et al., 2001). Specifically with the promotion of intermodality between aircraft and train, the European Commission aims to address some of the negative effects of transport (Eurocontrol, 2004b).

## The individual behavior in the choice of a service

One of the main objectives of social research is to determine the behavior of the consumer in the decision-making process and this is especially relevant when we are talking about services (McFadden, 2001b), whose supply is neither storable nor cumulative such as transport (Domencich and McFadden, 1975; de Dios Ortúza and Willumsen, 2001).

According to Ben-Akiva and Lerman (1985), an election can be considered as the result of a sequential decision-making process, which includes the following steps: definition of the problem of choice, generation of alternatives, evaluation of the attributes of each alternative, choice, and application of choice.

A specific theory of choice is therefore a collection of procedures and elements based on some general hypotheses. The elements that define the process of decision making are the decision maker and its characteristics, the alternatives or the determination of the options available for decision making, attributes, and decision rules, which describes the process used to choose a decision alternative (McFadden, 2001b; Ye et al., 2007; Martín et al., 2011; Chowdhury and Ceder, 2016; Houdek, 2016).

The decision-making or decision-making unit can be a person or a group of people, such as a family or an organization. If one considers a group of people as a decision maker we can ignore all the internal interactions within the group, and consider only the decisions of the group as a whole. In transport modeling, it has been customary to use aggregate models, which are calibrated with data that has been grouped or aggregated in some form (e.g., using average income per zone), although in the last decades a more disaggregated focus has been considered taking into account the decision unit, the individual (Domencich and McFadden, 1975). In this case, to explain the heterogeneity of preferences among decision makers at the individual level, a disaggregated model should include the socioeconomic characteristics of each decision maker, such as age, sex, and income (Collins and Chambers, 2005).

The analysis of individual decision-making requires not only the knowledge of what has been chosen, but also of what has not been chosen. Therefore, it is necessary to make hypotheses about the available options, or alternatives, that an individual considers during the election process. The set of considered alternatives is called the set of choice. We can differentiate two types of set of choice: continuous and discrete choice sets (Ben-Akiva and Lerman, 1985). The first case defines the set of choice taking into account all possible combinations of goods and services available to the individual^1^. On the contrary, a discrete choice set contains a finite number of mutually exclusive alternatives that can be specifically enumerated. The choice of a means of transport is a typical example of a choice of a discrete choice set^2^ (Taniguchi et al., 2014).

The identification of the list of alternatives is a complex process, since not all alternatives will be available all the time for all decision makers. Therefore, it is necessary to differentiate between the universal set of choice, which contains all possible alternatives and the set of choice. The latter is a subset of the universal set of choice available to a particular individual, since alternatives to the universal choice set not available to the individual are excluded.

Normally, to determine these alternatives, deterministic criteria of availability of alternatives are used (for example, having a driving license determines the availability of the alternative of car). In addition to availability, knowledge of the existence of an alternative is a very important factor for the choice made. In this sense, Ben-Akiva and Lerman define that the causes of an alternative is feasible depending on availability in the market, budgetary constraints, available time or other informal factors such as knowledge of the service of a given alternative (Ben-Akiva and Lerman, 1985; Chowdhury et al., 2015; Schakenbos et al., 2016).

Behavior aspects sow uncertainty in the production of alternatives and motivate the use of probabilistic models of choice generation that allow us to obtain the probability of each alternative in the universal set (Swait, 1984).

An attribute is a trait that characterizes an alternative, with a certain value for individuals. The neoclassical economic theory considers that the individuals choose an alternative, from the different amounts of the goods that are included in them. In contrast, the discrete choice theory considers each alternative as a set of attributes and in function of these the attraction of an alternative is expressed by an attribute vector, called the utility function (Ben-Akiva and Lerman, 1985). Moreover, each alternative has its own attributes, and these can be generic for all alternatives or specific for a specific alternative. An attribute is not necessarily an objective quantity that can be measured, but can derive from a subjective measurement from perceptions. Just as the characteristics of the decision maker are taken into account, the analyst must include the attributes of each alternative (Sandoval, 1994).

In decision making, the last of the elements is the decision rule, defined as the process used in decision making, to evaluate the information available on the attributes of each alternative, in the set of choice and to determine a single choice. There are numerous decision rules in the literature that can be grouped into the following four categories (Ben-Akiva and Lerman, 1985):

1. Dominance: one alternative is dominant over another, if it is better in at least one attribute and not worse in all the others. This rule in many situations can lead to the choice of several alternatives. To avoid this you can add to each attribute values that consider some better than others (minimum levels are defined for the election).
2. Satisfaction: for each attribute a level is given that serves as a criterion of satisfaction for the individual. If an alternative does not reach the minimum criterion in some of its attributes it can be eliminated as a choice.
3. Lexicographical rule: it is given that the attributes are ordered according to the level of importance. The decision maker chooses the alternative that is more attractive based on the most important attributes. You can also use this rule to eliminate the worst alternatives in each attribute in order of importance. The combination of the decision rule, in terms of satisfaction and lexicographic rules, is called “elimination by aspects”. This process begins with the most important attribute and eliminates the alternatives that do not reach the level of satisfaction. If two or more alternatives are available, the second most important attribute is continued and so on (Tversky, 1972).
4. Utility: This type of decision rule assumes that the attributes are measurable. This means that the attractiveness of an alternative is expressed by a vector of attribute values by means of a scalar, called utility. The individual will seek to maximize this utility (or minimize its costs).

These four decision rules, at the same time can be grouped into two types of behavior: compensatory or non-compensatory behavior. The compensatory type takes into account the set of all the attributes, so that changes in one or more attributes can be compensated by others, and in case of a decrease in one attribute can be compensated for an improvement in another, as is the case of the utility rule. In non-compensatory behaviors, rules or levels are defined to restrict the choice of some alternatives. Among these are the first three decision rules described (McFadden, 2001b).

The classical (neoclassical) and discrete-choice theories analyze individual consumer behavior (Ben-Akiva and Lerman, 1985), from a utility function that individuals must maximize, based on the analysis of revealed preferences, although with important differences between them, in the definition of decision rules and alternatives.

Most transport choice models have been based on this behavior (utility maximization), although other decision rules have been tried (Cantillo and Ortúzar, 2005), especially for the case of the declared preferences, where the choices that do not follow the principles of maximum utility are measured (Sælensminde, 2002; Rouwendal and Blaeij, 2004).

The mobility of people is a complex phenomenon, due to the large number of factors that influence the decisions of individuals. For this reason, it is necessary to analyze the displacements according to three interacting aspects a spatial approach, a social approach and a perspective approach (Rodríguez, 1991).

The spatial approach focuses on the different land use and the distribution of activities in space. This creates the necessity of the individual of the individual to move, depending on the location of the activities you want to perform.

The social approach refers to the fact that an individual's movements are exclusive of their characteristics and are therefore the result of the socioeconomic characteristics of the individual who performs it. Because of this it is necessary to have very present variables like the sex, the age, cultural level, income, health, etc.; for the analysis of the mobility.

Finally, we can speak of a perceptive approach, which refers to the image that the individual has formed. This image varies in each individual or group of people, leading to different assessments of the same data in decision making.

In the classic model a sequence of decisions of the individuals is done in four stages which are the generation of trips, zonal distribution, modal distribution, and assignment, but another sub-model has recently been included that is the last one mentioned, the election of the hour that gives place to the models of time distribution.

At present, this method is recognized to be too strict, since the decisions of the individuals are not taken following this sequence, but each sub-model depends on the type of the function of utility assumed to explain all these choices of trip. There are some current approaches that differ from this four-step sequential methodology and simultaneously address the stages of choice of frequency, destination, and mode of travel, but they are still at a level of research and have not been implemented. In addition, we must highlight models based on the activities of families or decision-making centers, which take into account constraints on budget and time choice (de Dios Ortúza and Willumsen, 2001) stand out.

## Methodological framework for modeling consumer choices

From an economic perspective, consumer theory is the economic modeling of the behavior of an economic agent that consumes goods and services. This theory relates preferences, indifference curves and budget constraints to consumer demand curves.

The fundamentals of the individual choice theory of the neoclassical model of consumer behavior are based on the fact that the individual chooses an amount of goods, which form their basket of goods, in order to maximize their utility which translates into their level of satisfaction, subject to their income restriction (Torres, 2008). Therefore, given a consumer whose preferences fulfill these assumptions, there is a deterministic utility function, U, which represents these preferences that will be ordered so that the individual will choose the alternative that gives him greater satisfaction^3^.

Given that consumer economic theory has been developed without taking into account the nature of the alternatives, some extensions have been made such as Strotz (1957, 1959), which analyzes consumer behavior by introducing the concept of “utility tree” (Strotz, 1957, p. 270). Muth (1966) proposes that the goods and services of the market are considered by the consumer as inputs of a domestic production function, whose production is the satisfaction of the consumer needs derived from consuming certain goods or Becker (1965) that extends the traditional theoretical formulation by adding the time constraint, in addition to the budget, to the production function.

Finally, one of the most important extensions is that one made by Lancaster (1966). This author considers three assumptions that break with the traditional approach and states. That (1) goods *per se* do not contribute to usefulness, but are the characteristics they possess, which provide usefulness to the consumer, (2) a good has more than one characteristic, and many of these characteristics can be shared by more than one good; (3) and through the combination of different goods different characteristics can be obtained from those corresponding to the separate goods. These assumptions represent the definition of utility in terms of the attributes of the goods.

Taking into account all these assumptions, consumer economic theory has important limitations to explain the consumer behavior, which justifies models based on Discrete Choice Theory. This reformulation of the behavior of the consumer will not occur until 1981, with the inclusion of consumer goods of a discrete nature (McFadden, 1981), with which the Theory of Discrete Choice begins, whose characteristics are that:

- The individual consumes continuous (or divisible) goods.
- The individual chooses between a set of mutually exclusive discrete alternatives (non-divisible goods).
- The discrete alternatives are represented by a feature vector Q_*j*_.

The problem that arises when using Discrete Choice Theory is that it represents a mechanism of deterministic choice and these deterministic election mechanisms do not fit the analysis of choice problems in real situations (Block and Marschak, 1960). In this line of study, this approach has been widely criticized, both in the field of psychology by Thurstone (1927), Luce (1959), and Tversky (1969), as in the field of economics by Quandt (1956) and McFadden (1981). These authors justify the inclusion of uncertainty in modeling, being the source of randomness determinant when specifying the different models.

Therefore probabilistic mechanisms of choice are required in order to analyze individual options. In order to do this, we use Probabilistic Election Theory, specifically to the Theory of Random Utility formalized by McFadden (1974, 1981), Domencich and McFadden (1975), and Manski (1977) and whose most recent developments are by (de Dios Ortúza and Willumsen, 2001).

The Random Utility Approach assumes that the individual always selects the most useful alternative, although the utilities are not known to the analyst and are treated as random variables. In mathematical terms, this is expressed by separating the total utility *U*_*in*_ into a deterministic component *V*_*in*_, called Systematic Utility and a random component ε_*i*_ that captures uncertainty, called Random Perturbation:

$$\begin{array}{l} U_{jq}=V_{jq}+\varepsilon_{jq} \end{array}$$

where:

*V*_*jq*_ is the systematic component that represents an appreciable part of the utility of the individual q to choose alternative *j*. Systematic utility depends on the attributes of the alternative and the socioeconomic characteristics of the person.

ε_*jq*_ is the random term that accounts for unobserved factors.

The individual chooses the most useful alternative. Alternative *i* is chosen, if and only if:

$$\begin{array}{l} P_{iq}=\;Prob\left(U_{iq}>\;U_{jq}\right)\forall j\neq i \end{array}$$

The analyst can only obtain the probability of choosing alternative *i* as:

$$\begin{array}{lll} P_{iq} & = & \;Prob\left(V_{iq}+\;\varepsilon_{iq}>\;V_{jq}+\;\varepsilon_{jq}\;\forall \;j\neq i\right)\\ P_{iq} & = & \;Prob\left(\varepsilon_{jq}-\varepsilon_{iq}<\;V_{iq}-\;V_{jq}\;\forall \;j\neq i\right) \end{array}$$

This is the probability that each random term ε_*nj*_ – ε_*ni*_, is less than an observed quantity *V*_*ni*_ − *V*_*nj*_, so it is a cumulative distribution function. From the density function *f*(ε*n*) we can calculate this probability as:

$$\begin{array}{l} P_{ni}=\;Prob\left(\varepsilon_{nj}-\varepsilon_{ni}<V_{ni}-\;V_{nj}\;\forall \;j\neq i\right)\\ \;=\int_{\varepsilon }\;I(\varepsilon_{nj}-\varepsilon_{ni}<V_{ni}-\;V_{nj}\;\forall \;j\neq i)f\left(\varepsilon_{n}\right)d\varepsilon_{n} \end{array}$$

Being *I (*·*)* a function that has a value of one if the term given in brackets is true (if the individual has chosen alternative *i*) and zero in another case. According to the different hypotheses formulated about the *distribution*, ε_*nj*_ will have a closed value of this integral (*Simple Logit or Hierarchical Logit*) or it will have to be evaluated numerically by simulation (*Probit or Mixed Logit*), and therefore the different Models of discrete choice (Train, 2003).

Defined the set of choice, we can proceed to define the utility function that will give rise to different types of models. The typology of this type of model is broad and can be classified (Medina, 2003 according to the number of alternatives of the endogenous variable (dichotomous response models and multiple response models) or according to the type of function to estimate the probability (Linear Probability Model, Logit Model, and Probit Model): Whether the alternatives are exclusive or incorporate ordinal information (models with non-ordered data and models with ordered data) and whether the regressors refer to aspects of individuals or to alternatives in non-ordered models (multinomial and conditional models).

For the theoretical justification of the models of discrete choice based on the Theory of Random Utility, we can find three types of models of discrete choice depending on the different hypotheses that are taken for the distribution of the random term. First the linear probability model, assuming a uniform distribution, then the Probit Model, assuming a normal distribution and in third place the Logit Model, assuming a logistic distribution.

The Linear Probability Model is the linear fit regression model that is applied to a binary dependent variable. This model is estimated by ordinary minimums to the square and is easy to estimate and interpret. The estimated parameters measure the predicted change in probability of success, vs. a unit increment of *X*_*i*_. Although there are problems in the estimation of the regression model when the endogenous variable is binary. The specific problems encountered with this model are *heteroskedasticity* of the perturbation term, the predicted probabilities are inconsistent, since it cannot be guaranteed that they are bounded between zero and one, the non-normality of the perturbation and the coefficient of Determination is not appropriate. Due to these problems, what is interesting is a model that reproduces properly the behavior of a probability function and the alternatives we find the Logit model and the Probit model, similar numerically. When the normal distribution is used as a probability function, the so-called Probit model is obtained, while the use of the logistic distribution provides the Logit model.

The main advantage of the Probit model is its ability to capture all correlations between alternatives, but its main problem is the complexity of its formulation, so there are very few applications that have been developed (Daganzo, 1979) and more than three alternatives are used for the calculation Simulation procedures (Train, 2003).

The logistic regression model, also called Logit models, is much more popular thanks to its analytical flexibility. Thus, the possible hypothesis for deriving some Logit models from the operation of individual choice is analyzed here: the most general case of interest, the Logit Multinomial model (McFadden, 1974), in its particular case, beyond binary situations; The Logit Hierarchical or Nested model (Williams, 1977) and the Logit Mixed model (McFadden and Train, 2000).

## Modeling the demand for services: application to the choice of transport services

Transport contributes significantly to the economic development and allows the market to function in a global way. It should be noted that most modes of transport do not affect society only in a positive ways, there are negative side effects such as congestion, noise and air pollution.

The promotion of intermodality using airplanes and trains is intended to solve some negative effects of transport, such as the impact of congestion on the environment, economy, safety and passengers (Eurocontrol, 2004b). For example, regarding to the access to the airport with the promotion of intermodality, beneficial effects are expected on the economy, especially in the regional economy, and on the environment are expected (Watkiss et al., 2001; Li and Hensher, 2012).

The estimation and internalization of external costs of transport have been important issues for research in the transport sector in the European Union. A Concern for the environment has grown over the last decade and the European Commission has raised the issue of internalization in several of its directives, such as the 1995 Green Paper (European Commission, 1995), the White Paper on 2001 (European Commission, 2001), and the Mid-Term Review in 2006 (European Commission, 2006). The latter two, underline the need for equitable and efficient pricing in terms of external costs. On the other hand, although the impact on the environment or congestion are indisputable, in the literature, there are some reports that try to quantify them, some of them are: (Intergovernmental Panel on Climate Change (IPCC), 1999; Aviation Environment Federation (AEF), 2000; INFRAS IWW, 2000, 2004; Whitelegg and Williams, 2001; IATA, 2003; Steer Davies Gleave, 2006; CE Delft, 2008) and (CE Delft, 2011).

In general, all of these investigations seek to establish pricing systems that capture the external costs associated with transport, so as to reduce negative impacts, in order to improve the efficiency of the transport system, to ensure fairness between modes and improve safety and sustainability.

In recent years, considerable progress has been made in the construction of transport demand models based on the theoretical principles of choice. Within random utility theory, it can be shown that the structure of the models depends on the perceived similarity between discrete choice alternatives. Moreover, this aspect can be interpreted mathematically in terms of the correlation between the components of random utility functions. The use of Logit-type disaggregated demand models allows us to determine the likelihood of choosing one way or another depending on the previously defined attributes, as well as quantifying the willingness to pay of these subjects based on the different characteristics previously given.

To do this, we must first determine which are the parameters that measure the utility of the services or that define the attributes of the service (Martín et al., 2011; Navarette and Ortuzar, 2013; Chowdhury et al., 2015; Schakenbos et al., 2016; Ettema et al., 2017). In order to select the mode of transport, the factors influencing the choice of mode can be classified into three groups (Eurocontrol, 2004a) by the characteristics of the mode of transport (vehicle availability and/or ownership, driving license, home structure, income), the trip characteristics (the mode of choice is heavily influenced by the purpose of the trip, time of travel), and the characteristics of transport infrastructures and services (relative travel time, relative monetary cost, comfort, and convenience, reliability and regularity, protection, and safety).

### Data sources

The information necessary for the estimation of these models is obtained through surveys of the mobility of the different individuals at a given moment (Martínez and Muro, 2011). Two types of surveys can be differentiated into revealed preferences and declared preferences.

Revealed preferences (RP) show data on the current behavior of individuals and give us information about their travel decisions. These provide us with descriptive information about the characteristics of the traveler on a particular route. Until the mid-1980s this type of data was the most used to model the transport demand. The main drawbacks to the use of this data in the modeling are the costs of the sample and its limited capacity to understand the behavior of the traveler, the observations of the actual elections may not have sufficient variability for the construction of good models. Because of this it is difficult to be able to detect the relative importance of certain factors on behavior; (de Dios Ortúza and Willumsen, 2001; Espino, 2003; Espino et al., 2006). For the Toledo-Madrid case, information is obtained on the type of mobility of travelers in the current situation with a socio-economic profile and mobility parameters (Martínez and Muro, 2014).

The stated preferences (SP) give information on the behavior of the individual before certain hypothetical situations raised by the researcher (Navarette and Ortuzar, 2013). Unlike RP data, which give information about the trips that an individual habitually makes, they inform about the trips that the individual would realize in certain conditions. The SPs started in the field of market studies and began to be applied to the field of transport demand analysis in the late 1970s. The possibility of designing SP experiments allows, theoretically, to solve the problems they present The RP (de Dios Ortúza and Willumsen, 2001). One of the problems presented by these models is that the analyst cannot assure that the individual performs what he has answered in an SP survey, so it is important to construct realistic alternatives and design comprehensible exercises to present the individual.

In an SP exercise, three elements can be mainly distinguished (Espino, 2003). The first are the situation in which the individual finds himself to declare his preferences which can be a real situation (a journey that is carried out at that moment) or hypothetical (a journey that would take place in the future given a series of conditions), and constitutes the context of the decision. Second, the alternatives that are usually hypothetical are selected, although some of them might exist today, they are presented in the exercise as a function of a set of attributes. Third, it defines how individuals can state their preferences. The most frequent techniques are the hierarchy of the answer, the punctuation of the alternatives and the choice between alternatives.

In the case of Toledo-Madrid, the different alternatives that the user has to carry out this journey are traveling by public transport, either by High Speed Train (HST) or by regular bus (BUS), or by private transport, Private vehicle (CAR).

The basic methodology for the presentation of the options of the declared preferences (SP) is the realization of an efficient design of the scenarios. Specifically, the declared preference questionnaire is designed following a time and price reduction scheme, in three blocks or scenarios, to determine the variation of the individuals' choice. The SP surveys are structured with two approaches: a first comparative approach between the bus and the HST and a second approach comparing the car and the HST. The scenarios are conformed with the variables of trip price, travel time, comfort and cost of parking, this last variable referred only to the case of the car.

The SP survey proposes a simulation exercise is exposed of alternative scenarios that layout the mobility in the future. For the elaboration of these scenarios the combination of price and travel time variables are used. Prior to the establishment of the options, a characterization of the services and infrastructures currently available for each mode of transport is made. This current quote will serve as a basis for calculating the different alternatives for the arrival to Madrid Airport from Toledo and choose the best.

Thus, the methodology followed was to calculate the price and average travel time of each mode of transport from the origin, which in this case is Toledo, to the destination in the current situation and compare it with the different hypothetical scenarios. Therefore, the scenarios that are presented in the SP questionnaires are calculated based on the average prices and times calculated in that route, and compared in each pair of transport modes (Muro, 2012).

Consequently, three route scenarios are established:

- Toledo-Airport without trans-shipment with the of the new infrastructure, making the trip in HST from Toledo to Madrid Airport making two stops, one at Atocha station and another at Chamartín station but without any transfer.
- Toledo-Airport with a transfer: with the new infrastructure, making the trip in HST from Toledo to Madrid Airport by making a stop at Atocha station and another stop with transshipment at Chamartín station.
- Toledo-Airport with two transfers with the use of the new infrastructure, making the trip in HST from Toledo to the Madrid Airport carrying out a stop with transshipment in the Atocha station and another stop with transshipment in the station of Chamartín.

The chosen bus and bus (CAR) optimum alternatives are fixed and compared in blocks of three, according to the three scenarios raised for the HST. The prices and travel times for the mode of transport bus and private vehicle are constant for all scenarios and are based on current values. The prices of the bus are calculated according to ticket prices and the prices of the private vehicle are calculated by applying a cost per kilometer journey that incorporates the maintenance of the vehicle, insurance, etc., adding to the cost of the toll. This price does not include parking costs since it is presented as a different variable measured in euros per day of parking.

In summary, for the calculation of these values of time and price, the following hypotheses have been taken into account:

1. Existence of a single ticket for the case of the HST, with information on possible transfer for all scenarios. Thus, the time of transfer between trains is minimized if necessary.
2. The travel time is composed of the sum of the time from the origin, the waiting and access times, and the travel time.
3. The prices related to the different stages of the trip in HST are calculated as variations of the base price, which is calculated based on the current trip.

From the econometric point of view, the main difference of both types of data (RP and SP) are the types of error that each present. The RP data suffer from errors in the measurement of independent variables and those of SP present errors in the dependent variable, motivated by the doubt about whether the individual will actually perform what he is declaring. The joint estimation of both types of data is based on the different errors mentioned and can be specified if we consider error terms with different variance. Ben-Akiva and Morikawa formulate mixed data estimates that combine revealed preferences and stated preferences with the aim of avoiding their disadvantages and taking advantage of different sources of information (Ben-Akiva and Morikawa, 1990).

### Specification and estimation

In order to explain the mathematical approach of the models of discrete choice applied to the case of transport choice, one can start with the assumption based on the choice of the individual between two available alternatives (binary response models), and then extend this assumption to the *j* alternatives available (multiple response models). The dichotomous response models have two categories and these usually indicate that an event has occurred, that some feature is present or that an option is chosen.

In the modeling of the choice of transport, the objective will be to analyze the choice of the different alternatives that the user has to make this journey, and these are for example to travel by public transport, either in High Speed Train (HST) or by regular bus (BUS), or by private transport, in a private vehicle (CAR).

As this study focuses on the probability of choosing a mode of transport, the available alternatives will be the high-speed train against that the bus and the car. This allows specifying and estimating binary logistic regression models that will serve as the basis for a first analysis of the goodness of the database. Thus, two models are specified that will have a functional form of binomial logistic regression model, whose alternatives will be HST-BUS and HST-CAR, respectively.

The functional form of the binomial logistic regression models with the HST-BUS / HST-CAR alternatives will be represented by:

$$\begin{array}{l} P\left(a\right)=P\left(U_{a}\geq U_{r}\right)=\;\frac{e^{\alpha V_{a}}}{e^{\alpha V_{r}}\;+e^{\alpha V_{a}}} \end{array}$$

Where *U* is the total utilities of the high-speed train (*a*) and bus/car (*r*), respectively.

If the deterministic utility component, *V*, is assumed to be linear with regard to the parameters, as in most disaggregated model approaches, then:

$$\begin{array}{l} P\left(a\right)=\frac{e^{\alpha \overset{-}{\beta }\;{\overset{-}{X}}_{a}}}{e^{\alpha \overset{-}{\beta }\;{\overset{-}{X}}_{r}}\;+e^{\alpha \overset{-}{\beta }\;{\overset{-}{X}}_{a}}} \end{array}$$

where:

$\overset{-}{\beta }$, is the vector of coefficients.

$\overset{-}{X}$, is the vector of independent variables.

So if we have two alternatives, for example the binomial Logit model will be represented

$$\begin{array}{l} Prob\left(Y_{i}=1\right)=\frac{1}{1+e^{-\left(\alpha +\beta_{k}{X_{k}}_{i}\right)}}=\frac{e^{\alpha +\beta_{k}{X_{k}}_{i}}}{1+e^{\alpha +\beta_{k}X_{ki}}} \end{array}$$

$$\begin{array}{l} Prob\left(Y_{i}=1\right)=\frac{1}{1+e^{-\alpha -\beta_{1}{X_{1}}_{i}-\beta_{2}X_{2i}}}=\frac{e^{\alpha +\beta_{1}{X_{1}}_{i}+\beta_{2}X_{2i}}}{1+e^{\alpha +\beta_{1}X_{1i}+\beta_{2}X_{2i}}} \end{array}$$

If we suppose that α = 1, since in the case linear utilities with regard to the parameters, the parameter α cannot be distinguished from the general scale of the β.

$$\begin{array}{l} P\left(a\right)=\;\frac{e^{\overset{-}{\beta }\bar{X_{a}}}}{e^{\overset{-}{\beta }\bar{X_{r}}}+e^{\overset{-}{\beta }\bar{X_{a}}}\;} \end{array}$$

Taking the logarithm on both sides, from the equation above, we obtain a formulation that is suitable for applying linear regression techniques:

$$\begin{array}{l} ln\frac{P\left(a\right)}{1-P\left(a\right)}=\bar{\beta \;}\left(\bar{X_{a}}-\bar{X_{r}}\right) \end{array}$$

Since the probability of choosing the alternative *r* is given by: *P*(*r*) = 1 − *P*(*a*)

The final functional form of the model with an additive linear utility function is as follows:

$$\begin{array}{l} ln\frac{P\left(a\right)}{P\left(r\right)}=\bar{\beta \;}\left(\bar{X_{a}}-\bar{X_{r}}\right) \end{array}$$

The main explanatory variables that are considered in the transport demand analysis are, differential between the attributes and the socioeconomic variables (de Dios Ortúza and Willumsen, 2001; Cokasova, 2003; Espino, 2003; Eurocontrol, 2005a; Steer Davies Gleave, 2006; Muro, 2012) which are:

1. Attribute variables:
  - Travel cost:
    The price variable price measured in euros will be the price paid for each of the means of transport used as public transport, and for the private vehicle, the cost of gasoline based on the kilometers traveled, including the cost of the amortization of the vehicle (insurance, maintenance, etc.). The travel cost variable represent the price paid, in euros, for the route according to the form of transport chosen. In addition to the private car, the price of the car is different, so the total cost of the car will be the sum of the cost of the trip plus the cost of parking.
  - Travel time:
    The total travel time is one of the most important factors that the traveler takes into account for choosing a mode of transport (Steer Davies Gleave, 2006). It is therefore a major factor to increase the demand, as travel time is reduced (IATA, 2003). The variable of travel time measured in minutes can be represented as the total time of the trip or by the time dedicated to each of the stages of the journey, such as waiting time, transfer, etc. The variable time considered in this study is the total time spent on the trip done by the chosen mode of transport.
  - Comfort:
    This variable measures the level of service and is represented by dummy variables, which include values whose coding contains the different levels of service perceived by the individual. In this case, comfort tries to measure how the individual perceives the transshipments that are presented as hypothetical scenarios of the future services of the HST to Madrid airport (for the rest of the alternatives they would be the current travel options). Comfort is the dummy variable defined as the number of transfers chosen for the alternative train. So that in the first scenario, with no transfers, the comfort is 1; in the second scenario, with one trans for, the comfort is 2; and the third scenario, with two transfers the comfort is 3. If you do not choose the HST option the comfort level will be 0.
2. Socioeconomic variables:
  -Sex:
    The variable sex is included as a dichotomous dummy variable, to analyze the influence of them in the decisions
  - Level of income was specified by the individual corresponding to the monthly salary range was divided into five categories being the first of an income of up to 600 € then income in the range of 601 to 1,000 €, following that in the range of 1,001 to 2,000 next in the range of 2,001 to 3,000 € and last above 3,000.
  - Age:
    This variable is expressed in four ranges: the value 1 being from 18 to 33 years; 2 from 34 to 49 years; 3 from 50 to 64 years, and 4 over 65 years. So we are in the specification of three dummy variables that represent value one when it meets that range and zero otherwise.

The utility specification is expressed as a Logit Binomial model, to measure the choice of mode of transport between the HST/BUS (*MNL1*) alternatives, in the Toledo-Madrid corridor (Madrid Airport):

Utility HST1: U_HST1_ = ASC_HST1_ + θ_P_P_HST1_ + θ_T_T_HST1_

Utility BUS: U_BUS_ = ASC_bus_ + θ_P_P_bus_ + θ_T_T_bus_ + ∑θ_Cj_C

Being,

*ASC*_*HST*1_ and ASC_*bus*_, the specific constants of each alternative, in which ASC_*HST*1_ is fixed.

θ_*P*_, θ_*T*_, and θ_*Cj*_ are the parameters associated to each of the explanatory variables. In this specification, the two most important variables (cost, θ_*P*_ and time, θ_*T*_), where *C*, refers to all socioeconomic variables and characteristics of travel such as income, sex, age, motive travel, and comfort.

*C*_*j*_, is a generic variable that represents each and every one of the variables not explicitly collected but mentioned above, where *j* refers to *S*, sex; *ING* to income; and *COM*, comfort depending on the transfers.

*P*_*HST*_, *P*_*bus*_, is the price of alternatives HST1 and Bus, respectively.

*T*_*HST*_, *T*_*bus*_, is the travel time of the alternatives HST1 and Bus, respectively.

The specification of the utilities of the two alternatives of the HST-CAR Model (*MNL2*) will be:

Utility HST2: U_HST2_ = ASC_HST2_ + θ_P_P_HST2_ + θ_T_T_HST2_

Utility CAR: U_CAR_ = ASC_car_ + θ_P_P_car_ + θ_T_T_car_ + θ_PA_PA_car_ + ∑θ_Cj_C_j_

Being, *ASC*_*HST*2_; ASC_*car*_, the specific constants of each alternative.

θ_*P*_*;* θ_*T*_*;* θ_*PA*_, and θ_*Cj*_ are the parameters associated to each of the explanatory variables.

*C*_*j*_, is a generic variable that represents each and every one of the variables not explicitly collected but mentioned previously.

*P*_*HST*_, *P*_*car*_, is the price of alternatives HST2 and CAR, respectively.

*T*_*HST*_, *T*_*car*_, is the travel time of alternatives HST2 and CAR, respectively.

The results of the binomial model estimates are shown in Tables 1, 2 (Bierlaire, 2003)^4^.

**Table 1:** Estimated logit binomial model results MNL1 (HST1-BUS).

**Models**	**MNL1**	**MNL1.1**			**MNL1.2**			**MNL1.3**			**MNL1.4**			**MNL1.5**			**MNL1.6**			**MNL1.7**		
		**Value**	***T-*****test**		**Value**	***T-*****test**		**Value**	***T-*****test**		**Value**	***T-*****test**		**Value**	***T-*****test**		**Value**	***T-*****test**		**Value**	***T-*****test**	
Constant Alt. HST1	ASC1	–	–	–	–	–	–	–	–	–	–	–	–	–	–	–	–	–	–	–	–	–
Constant Alt. BUS	ASC2	−0.039	−0.240		0.587	2.550	^**^	0.780	4.170	^***^	−0.159	−0.940		0.069	0.400		0.629	3.200	^***^	0.271	0.000	
Total travel cost	θ_PT_	−0.089	−6.140	^***^	−0.091	−5.490	^***^	−0.091	−6.240	^***^	−0.089	−6.150	^***^	−0.091	−6.230	^***^	−0.092	−6.240	^***^	−0.092	−6.250	^***^
Travel Time	θ_T_	−0.038	−5.400	^***^	−0.394	−5.490	^***^	−0.039	−5.480	^***^	−0.038	−5.410	^***^	−0.039	−5.470	^***^	−0.036	−2.500	^**^	−0.039	−5.490	^***^
Sex	θ_S_	−	–		0,115	0,080		–	–	–	–	–	–	–	–	–	–	–	–	0,271	0,000	
Income	θ_I_	–	–		−0.293	1.440		−0.302	0.032		–	–	–	–	–	–	−0.216	−4.610	^***^	−0.205	−4.320	^***^
Time_Sex	θ_TS_	–	–	–	–	–	–	–	–	–	0.002	3.280	^***^	0.001	2.220	^**^				−0.001	0.000	
Cost_Income	θ_PTI_	–	–	–	–	–	–	–	–	–	–	–	–	−0.082	−8.210	^***^	−0.036	−2.500	^**^	−0.071	−2.550	^**^
R-Squared	*R*^2^	0.059			0.083			0.082			0.006			0.080			0.084			0.084		
Adjusted R-Squared	${\bar{R}}^{2}$	0.057			0.080			0.080			0.062			0.080			0.081			0.081		
Log-likelihood	I(θ)	−1902,255			−1854,424			−1855,462			−1896,862			−1860,499			−1852,315			−1851,139		
Sample	N	2916			2916			2916			2916			2916			2916			2916		
Willingness to pay	VOT	0.431			4.311			0.430			0.430			0.430			0.397			0.430		

Value is the estimated coefficient for each of the explanatory variables included in the model. T-test:

^*^ 90%,

** 95%, and

*** *99% level of significance. It is the statistic that allows to contrast the null hypothesis of individual non-significance of each of the variables included in the model, (the contrast is performed through the level of significance associated with that statistic, so that if the level of significance is greater than 0.05 the variable is not statistically significant).The MNL1 Models are the results of the estimation with the data of the alternatives HST1 and BUS. The MNL1.1, MNL1.2, MNL1.3, MNL1.4, MNL1.5, MNL1.6, and MNL1.7 models are variants of specified models with different explanatory variables and the different specifications represent a robustness check*.

**Table 2:** Estimated binomial model results MNL2 (HST2-CAR).

**Models**	**MNL2**	**MNL2.1**			**MNL2.2**			**MNL2.3**			**MNL2.4**			**MNL2.5**			**MNL2.6**			**MNL2.7**		
		**Value**	***T-*****test**		**Value**	***T-*****test**		**Value**	***T-*****test**		**Value**	***T-*****test**		**Value**	***T-*****test**		**Value**	***T-*****test**		**Value**	***T-*****test**	
Constant Alt. HST1	ASC1	–	–	–	–	–	–	–	–	–	–	–	–	–	–	–	–	–	–	–	–	–
Constant Alt. CAR	ASC4	−4.2	−12.92	^***^	−3.85	−10.62	^***^	−4.2	−12.92	^***^	−4.15	−9.78	^***^	−5.00	−13.3	^***^	−4.14	−12.6	^***^	0.768	0.34	
Travel cost	θ_P_	–	–	–	0.0219	1.34		–	–	–	–	–	–	–	–	–	–	–	–	–	–	–
Parking cost	θ_PA_	–	–	–	−0.0128	−3.92	^***^	–	–	–	–	–	–	–	–	–	–	–	–	–	–	–
Total travel cost	θ_PT_	−0.0114	−3.58	^***^	–	–	–	−0.0114	−3.58	^***^	−0.011	−3.41	^***^	−0.012	−3.75	^***^	−0.0119	−3.58	^***^	−0.0101	−2.97	^***^
Travel time	θ_T_	−0.117	−9.71	^***^	−0.0879	−4.83	^***^	−0.117	−9.71	^***^	−9.72	0.0121		−0.119	−9.77	^***^	−0.12	−9.86	^***^	−0.108	−8.53	^***^
Sex	θ_S_	–	–	–	–	–	–	–	–	–	−0.529	−4.1	^***^	–	–	–	–	–	–	−5.43	−2.58	^***^
Income	θ_I_	–	–	–	–	–	–	–	–	–	0.183	3.41	^***^	0.237	4.51	^***^	–	–	–	0.277	3.06	^***^
Time_Sex	θ_TS_	–	–	–	–	–	–	–	–	–	–	–	–	–	–	–	−0.0081	−4.27	^***^	0.0718	2.34	^**^
Cost_Income	θ_PTI_	–	–	–	–	–	–	–	–	–	–	–	–	–	–	–	0.0027	0.001		−0.00295	−1.3	
R-Squared	*R*^2^	0.524			0.525			0.524			0.533			0.529			0.53			0.534		
Adjusted R-Squared	${\bar{R}}^{2}$	0.522			0.523			0.522			0.530			0.527			0.528			0.531		
Log-likelihood	I(θ)	−1033,143			−1030,897			−1033,143			−1013,763			−1022,498			−1018,718			−1009,741		
Sample	N	3129			3129			3129			3129			3129			3129			3129		
Willingness to pay	VOT	10.3			−4.02			10.3			10.8			9.91			10.1			10.7		

Value is the estimated coefficient for each of the explanatory variables included in the model. T-test: ^*^ 90%,

** 95% and

*** *99% level of significance. It is the statistic that allows to contrast the null hypothesis of individual non-significance of each of the variables included in the model, (the contrast is performed through the level of significance associated with that statistic, so that if the level of significance is greater than 0.05 the variable is not statistically significant).The MNL2 Models are the results of the estimation with the data of the alternatives HST2 and CAR. The MNL2.1, MNL2.2, MNL2.3, MNL2.4, MNL2.5, MNL2.6, and MNL2.7 models are the same as the previous variants of the original and the different specifications represent a robustness check*.

The MNL1 models Table 1 are the result of the estimation of the data of the alternatives HST1 and BUS. The MNL1.1, MNL1.2, MNL1.3, MNL1.4, MNL1.5, MNL1.6, and MNL1.7 models are variants of specified models with different explanatory variables. In general, these variables are statistically significant, as it shows in Table 1, and with correct signs (negative values for its inverse relationship).

The results of the first binomial models show low determination coefficients, which are a reflection of the inadequate fit of the Logit Binomial model at around 1% in the case of the HST-BUS alternatives (MNL1.1: 0.057; MNL1.2: 0.080; MNL1.6: 0.81). This can be caused by the lower amount of people who chose the BUS option vs. the HST option.

The MNL2 models Table 2 are the results of the estimation of the data of the alternatives HST1 and BUS. The MNL1.1, MNL1.2, MNL1.3, MNL1.4, MNL1.5, MNL1.6, and MNL1.7 models are variants of specified models with different explanatory variables. In general, these variables are statistically significant, as it is shown in Table 2, and with correct signs (negative values for its inverse relationship), except in the MNL2.2 where we can find the travel cost with positive values. For this reason this model is nullified and in the final models only the cost of the total trip is included.

The MNL2 models (Table 2) are the results of the estimation with the data of the alternatives HST2 and CAR. The MNL2.1, MNL2.2, MNL2.3, MNL2.4, MNL2.5, MNL2.6, and MNL2.7 models are the same as the previous variants of the original. These models show determination coefficients around 53% (MNL2.1: 0.522, MNL2.3: 0.522; MNL2.7: 0.531).

From the results of the estimated models we proceed to calculate the payment arrangements of the users. These payment arrangements are calculated as the quotient of the parameters estimated for time and price, so they are interpreted as the willingness to pay or waiting to save in travel time (Espino et al., 2006).

The provisions for the payment of the binomial HST-BUS, fluctuate between 0.397 and 0.431, which means that individuals would be willing to pay from 23 to 25 € to save an hour of travel.

In the case of Logit Binomial HST-CAR models, Table 2, the results are very high being 600 € per hour what is saved in travel time. This may be motivated by individuals who answered in a lexicographic form, in which regardless the options, the users have chosen car, although this was but in all the options and by the difficulty of capturing the usual users of the car.

Next, if we assume that there are three available alternatives of choice for the individual in this path, a model is proposed with three alternatives, which are HST, bus and car, whose functional form will be that of a multinomial logistic regression model.

The specification is based on the approach of four alternatives, two from each of the sub-databases:

Utility HST1: U_HST1_ = ASC_HST1_ + θ_P_P_HST1_ + θ_T_T_HST1_

Utility BUS: U_BUS_ = ASC_BUS_ + θ_P_P_BUS_ + θ_T_T_BUS_

Utility HST2: U_HST_ = ASC_HST2_ + θ_P_P_HST2_ + θ_T_T_HST2_

Utility CAR: U_CAR_ = ASC_CAR_ + θ_P_P_CAR_ + θ_T_T_CAR_ + θ_PA_PA_CAR_ + ∑θ_Cj_C_j_

The joint treatment of both databases requires, in the specification of the multinomial model, a specific treatment and equal to the problematic applicable to Mixed data (RP and SP).

Thus, the Logit Multinomial (MNL3) model with the HST-BUS-CAR alternatives will have the form:

The probability of choosing each of the alternatives will be:

$$\begin{array}{l} Prob\left(Y_{i}=1\right)=\frac{1}{1+e^{\alpha_{2}+\beta_{12}X_{1i}+\beta_{22}X_{2i}}+e^{\alpha_{3}+\beta_{13}X_{1i}+\beta_{23}X_{2i}}} \end{array}$$

$$\begin{array}{l} Prob\left(Y_{i}=2\right)=\frac{e^{\alpha_{2}+\beta_{12}X_{1i}+\beta_{22}X_{2i}}}{1+e^{\alpha_{2}+\beta_{12}X_{1i}+\beta_{22}X_{2i}}+e^{\alpha_{3}+\beta_{13}X_{1i}+\beta_{23}X_{2i}}} \end{array}$$

$$\begin{array}{l} Prob\left(Y_{i}=3\right)=\frac{e^{\alpha_{3}+\beta_{13}X_{1i}+\beta_{23}X_{2i}}}{1+e^{\alpha_{2}+\beta_{12}X_{1i}+\beta_{22}X_{2i}}+e^{\alpha_{3}+\beta_{13}X_{1i}+\beta_{23}X_{2i}}} \end{array}$$

Therefore, a multinomial Logit model (*MNL3*) is specified, with sub-bases 1 and 2, so that a specification is made with the mixed data processing with different errors and therefore variances, so that ε is the stochastic error Of the HST-BUS data (Base 1) and η the data of the base HST-CAR (Base 2). It can be expressed as: $\;\sigma_{\varepsilon }^{2}=\mu^{2}\;.\;\sigma_{\eta }^{2}$, where μ is an unknown parameter.

So the utilities of both databases are expressed

$$\begin{array}{lll} U_{j}^{B1} & = & \;V_{j}^{B1}+\;\varepsilon_{j}=\;\theta .\;X_{j\;}^{B1}+\;\alpha .\;Y_{j\;}^{B1}+\;\varepsilon_{j}\\ \mu U_{j}^{B2} & = & \;\mu \left(V_{j}^{B2}+\eta_{j}\right)=\;\mu \;\left(\theta .\;X_{j\;}^{B2}+\omega Z_{j}^{B2}+\eta_{j}\right) \end{array}$$

where:

θ, α, and *w* are the parameters to be estimated.

$X_{j}^{BI}$ and $X_{j}^{B2}$ are common attributes of alternative *j* for databases 1 and 2, respectively, while $Y_{j}^{B1}$ and $Z_{j}^{B2}$ are non-common attributes of alternative *j* for each data set.

By multiplying the utility function of the Base 2 data by the unknown parameter μ, what is obtained is that the stochastic error of this type of data has the same variance as the data of Base 1.

Taking into account these utility functions we are allowed to homogenize the type of error homogenized, due to the multiplication of the SP parameters where by multiplying the utility function of the sub-base data 2 by the unknown parameter μ, we ensure that the stochastic error of this type of data has the same variance as the data of sub-base 1.

In mixed data, it is assumed that SP data should have more noise than RP data. If this is the case, the value of μ, that is known as the *scale coefficient* of the model, will be between 0 and 1. If the value is >1 it would indicate that the data with the highest noise level are those of RP. In our case and due to the errors detected, we assume that base 2 (HST-CAR) will have more noise (Ben-Akiva and Morikawa, 1990)^5^.

Taking into account the mixed data (*MNL3.1*) the specification of the model would be: U_HST1_ = OP ^*^ (ASC_HST1_ + BETA1 ^*^P_HST1_ + BETA2 ^*^T_HST1_) + (1-OP) ^*^μ^*^ (ASC_HST2_ + BETA1 ^*^P_HST2_ + BETA2 ^*^T_HST2_) U_BUS_ = OP ^*^ (ASC_BUS_ + BETA1 ^*^ P_BUS_ + BETA2 ^*^T_BUS_) + (1-OP)^*^ μ^*^ (ASC_BUS_ + BETA1 ^*^P_BUS_ + BETA2 ^*^T_BUS_) U_HST2_ = OP ^*^ (ASC_HST1_ + BETA1 ^*^P_HST1_ BETA2 ^*^T_HST1_) + (1-OP) ^*^μ^*^ (ASC_HST2_ + BETA1 ^*^P_HST2_ BETA2 ^*^T_HST2_) U_CAR_ =OP ^*^ (ASC_CAR_ + BETA1 ^*^ CT_CAR_ + BETA2 ^*^T_CAR_) + (1-OP) ^*^μ^*^ (ASC_*CAR*_ + BETA1 ^*^ PT_CAR_ + BETA2 ^*^T_CAR_)

We proceeded to estimate multinomial models with three and four alternatives. The results of the estimation of the models are shown in Tables 3, 4 (Bierlaire, 2003), for the multinomial simple models (MNL) with four and three alternatives, respectively.

**Table 3:** Estimated MNL results with four alternatives and mixed data.

**Models**	**MNL3**	**MNL3.1**		
		**Value**	***T-*****test**	
Constant Alt. HST1	ASC1	–	–	–
Constant Alt. BUS	ASC2	−0,011	−0.06	
Constant Alt. HST2	ASC3	–	–	–
Constant Alt. CAR	ASC4	−20.4	−1.85	
Total travel cost	θ_PT_	−0.089	−6.14	^***^
Travel time	θ_T_	−0.039	−5.55	^***^
Theta	μ	0.083	2.23	^*^
*R*-Squared	*R*^2^	0.289		
Adjusted R-Squared	${\bar{R}}^{2}$	0.287		
Log- likelihood	I(θ)	−2981,137		
Sample	N	6045		
Willingness to pay	VOT	0.438		

Value is the estimated coefficient for each of the explanatory variables included in the model. T-test:

* *90%*,

^**^ 95% and

*** *99% level of significance. It is the statistic that allows to contrast the null hypothesis of individual non-significance of each of the variables included in the model, (the contrast is performed through the level of significance associated with that statistic, so that if the level of significance is greater than 0.05 the variable is not statistically significant)*.

**Table 4:** Results of MNL models estimated with three alternatives and with scaled data.

**Models**	**MNL4**	**MNL4.1**			**MNL4.2**			**MNL4.3**			**MNL4.4**			**MNL4.5**			**MNL4.6**		
		**Value**	***T-*****test**		**Value**	***T-*****test**		**Value**	***T-*****test**		**Value**	***T-*****test**		**Value**	***T-*****test**		**Value**	***T-*****test**	
Constant Alt. HST	ASC1	1.65	9.09	^***^	–	–	–	–	–	–	–	–	–	–	–	–	–	–	–
Constant Alt. BUS	ASC2	1.49	0.251		−0.011	−0.07		–	–	–	−2.71	−8.33	^***^	–	–	–	−2.71	−8.33	^***^
Constant Alt. CAR	ASC3	–	–	–	−20.4	−20.42	^***^	–	–	–	−28.7	−19.86	^***^	–	–	–	−28.7	−19.86	^***^
Total travel cost	θ_PT_	−0.011	−3.68	^***^	−0.089	−6.59	^***^	−0.314	−28.15	^***^	−0.101	−7.44	^***^	–	–	–	–	–	–
Travel cost	θ_P_	–	–	–	–	–	–	–	–	–	–	–	–	−0.108	−28.15	^***^	−0.0935	−6.5	^***^
Parking cost	θ_PA_	–	–	–	–	–	–	–	–	–	–	–	–	−0.499	−29.93	^***^	−0.154	−3.91	^***^
Travel time	θ_T_	−0.012	−3.56	^***^	−0.039	−5.8	^***^	−0.112	−26.97	^***^	0.00303	0.38		−0.0468	−9.55	^***^	0.00613	0.74	
Commodity	θ_TRA_	–	–	–	–	–	–	0.099	−2.74	^***^	−0.723	−9.58	^***^	−0.113	−3.1	^***^	−0.722	−9.55	^***^
R-Squared	*R*^2^	0.285			0.289			0.229			0.3			0.281			0.3		
Adjusted R-Squared	${\bar{R}}^{2}$	0.285			0.288			0.229			0.299			0.281			0.299		
Log-likelihood	I(θ)	−2993,949			−2981,137			−3228,943			−2931,106			−3010,573			−2930,055		
Sample	N	6045			6045			6045			6045			6045			6045		
Willingness to pay	VOT	1.063			0.438			0.356			−0.0301			0.435			−0.0656		
Willingness to pay	VOTRA	–			–			1.356			7.19			1.05			7.73		

Value is the estimated coefficient for each of the explanatory variables included in the model. T-test: ^*^ 90%, ^**^ 95% and

*** *99% level of significance. It is the statistic that allows to contrast the null hypothesis of individual non-significance of each of the variables included in the model, (the contrast is performed through the level of significance associated with that statistic, so that if the level of significance is greater than 0.05 the variable is not statistically significant). Estimated model with three alternatives without scaled data that serves to verify how successive models with scaled data improve the results*.

Subsequently, models that combine the two databases have been estimated applying the specific problem of mixed data (Ben-Akiva and Morikawa, 1990), estimating Multinomial Logit models, with three and four alternatives. Once detected that the scale value of mixed data is significant, which means that there are differences in errors in the two databases, we start scaling the data, verifying that the results improve with the scaled data. From this moment on when estimating the Multinomial Logit models, the data of the sub-base 2 are scaled by this calculated coefficient in the model MNL3.1.

In Table 3 it can be verified that the variable Theta (μ) can be verified to be statistically significant. For this reason, the database 1 must be scaled in order to be able to establish alternatives HST1 and HST2 as a single HST alternative. Thus, the three estimated alternatives models will have the scaled data. (Tables 4, **6**).

In Table 4 the estimated model is shown with three alternatives. From the MNL4.1 model with three alternatives without scaled data we can verify how in subsequent models with scaled data the results are improved in terms of Pseudo-R2 (ρ2). Therefore, from the scaled data the proposed specifications are estimated with three alternatives of Multinomial Logit and Mixed Logit models. From each of these results shown the provisions to be paid are calculated as the quotient of the estimated parameters for time and price.

Finally, the specification of a *Logit Mixed model* (ML), of fixed parameters, is carried out. The *Logit Mixed model with error components* is used when analyzing arbitrary substitutability or correlation patterns. In this case the utility of the alternative *i* is specified as:

$$\begin{array}{l} U_{iq}=\alpha \prime x_{iq}+{\mu \prime }_{q}z_{iq}+\varepsilon_{iq} \end{array}$$

where:

$$\begin{array}{l} x_{iq}\text{ and }z_{iq},\text{ are vectors of observed variables}. \end{array}$$

α, is a vector of fixed coefficients and represents the parameter vector of the explanatory variables of alternative *i*.

μ_*q*_ and ε_*iq*_, are independent, being μ_*q*_, is a vector of random coefficients with mean 0 and covariance W y ε_*iq*_, are random variables, iid Gumbel(0, β).

With this formulation the random term is:

$$\begin{array}{l} \eta_{iq}={\mu \prime }_{q}z_{iq}+\varepsilon_{iq} \end{array}$$

It can be verified that with this formulation there is correlation between any pair of alternatives:

$$\begin{array}{l} \mathrm{cov}\left(\eta_{iq},\eta_{iq}\right)=E\left[{\mu \prime }_{q}z_{iq}+\varepsilon_{iq}\right]E\left[{\mu \prime }_{q}z_{jq}+\varepsilon_{jq}\right]\\ ={z\prime }_{iq}Wz_{iq}+\sigma_{\varepsilon }^{2}I\neq 0\text{si}z_{iq}\neq 0 \end{array}$$

The probability of choice can be obtained in a similar way to the previous case, conditioned by the value of μ_*q*_. Due to the fact that there is no guarantee of being able to solve the integral, the probability is obtained by simulation.

Both models (of random parameters and error components) are equivalent when the vector parameter β breaks clown into its mean α plus the deviations μ_*q*_ (β_*q*_ = α + μ_*q*_*)*. Reciprocally, if *z*_*iq*_ = *x*_*iq*_ the error component model is equivalent to a random parameter model: and if *z*_*iq*_ = *x*_*iq*_, we would obtain a random parameter model with fixed coefficients for *x*_*iq*_ and random coefficients with zero mean for *z*_*iq*_.

The specification of a *Logit Mixed* (*ML*) model, with a panel effect to analyze the correlation between variables, using a fixed effects model, where four alternatives are specified first and further on three are specified.

First, a Logit Mixed model (*ML5.1*) is proposed to determine the panel effect, with mixed data and with the same error component for the two databases, so that the model will be:

$$\begin{array}{l} {\text{U}}_{\text{HST1}}={\text{OP}}^{*}({\text{ASC}}_{\text{HST1}}+{\text{BETA}1}^{*}{\text{P}}_{\text{HST1}}+{\text{BETA}2}^{*}{\text{T}}_{\text{HST1}})\\ \;+{\left(\text{1-OP}\right)}^{*}\mu^{*}({\text{ASC}}_{\text{HST2}}+{\text{BETA}1}^{*}{\text{P}}_{\text{HST2}}+\\ \;{\text{BETA}2}^{*}{\text{T}}_{\text{HST2}})\\ {\text{U}}_{\text{BUS}}={\text{OP}}^{*}({\text{ASC}}_{\text{BUS}}+{\text{BETA}1}^{*}\\ \;P_{\text{BUS}}+{\text{BETA}2}^{*}{\text{T}}_{\text{BUS}})+\text{ZERO}[\text{ SIGMA1}]+\;)\;\\ \;{\left(\text{1-OP}\right)}^{*}\mu^{*}({\text{ASC}}_{\text{BUS}}+{\text{BETA}1}^{*}{\text{P}}_{\text{BUS}}+{\text{BETA}2}^{*}\\ \;{\text{T}}_{\text{BUS}}+\text{ ZERO}[\text{ SIGMA1}]\text{ )}\\ {\text{U}}_{\text{HST2}}={\text{OP}}^{*}({\text{ASC}}_{\text{HST1}}+{\text{BETA}1}^{*}{\text{P}}_{\text{HST1}}{\text{BETA}2}^{*}{\text{T}}_{\text{HST1}})\\ \;+\;{\left(\text{1-OP}\right)}^{*}\mu^{*}({\text{ASC}}_{\text{HST2}}+{\text{BETA}1}^{*}{\text{P}}_{\text{HST2}}\\ \;+\;{\text{BETA}2}^{*}{\text{T}}_{\text{HST2}})\\ {\text{U}}_{\text{CAR}}={\text{OP}}^{*}({\text{ASC}}_{\text{CAR}}+{\text{BETA}1}^{*}{\text{CT}}_{\text{CAR}}+{\text{BETA}2}^{*}{\text{T}}_{\text{CAR}}\\ \;+\text{ZERO}[\text{ SIGMA1}]\;)\;+\\ \;{\left(\text{1-OP}\right)}^{*}\mu^{*}({\text{ASC}}_{\text{CAR}}+{\text{BETA}1}^{*}{\text{PT}}_{\text{CAR}}\\ \;+{\text{BETA}2}^{*}{\text{T}}_{\text{CAR}}+\text{ZERO}[\text{ SIGMA1}]\text{ )} \end{array}$$

Being: OP = 1 with values of sub-base 1 and 0 for values of sub-base 2 (OP-1 = Base 2)

And later it is specified with different errors for each base (ZERO_SIGMA1 and ZERO_SIGMA2)

$$\begin{array}{l} {\text{U}}_{\text{HST1}}={\text{OP}}^{*}({\text{ASC}}_{\text{HST1}}+{\text{BETA}1}^{*}{\text{P}}_{\text{HST1}}+{\text{BETA}2}^{*}{\text{T}}_{\text{HST1}})\\ \;{\text{U}}_{\text{BUS}}={\text{OP}}^{*}({\text{ASC}}_{\text{BUS}}+{\text{BETA}1}^{*}{\text{P}}_{\text{BUS}}+{\text{BETA}2}^{*}{\text{T}}_{\text{BUS}})\\ \;+\text{ ZERO}[\text{ SIGMA1}]\text{ )}\\ {\text{U}}_{\text{HST2}}={\left(\text{1-OP}\right)}^{*}\mu^{*}({\text{ASC}}_{\text{HST2}}+{\text{BETA}1}^{*}{\text{P}}_{\text{HST2}}\\ \;+{\text{BETA}2}^{*}{\text{T}}_{\text{HST2}})\\ {\text{U}}_{\text{CAR}}={\left(\text{1-OP}\right)}^{*}\mu^{*}({\text{ASC}}_{\text{CAR}}+{\text{BETA}1}^{*}{\text{PT}}_{\text{CAR}}\\ \;+{\text{BETA}2}^{*}{\text{T}}_{\text{CAR}}+\text{ZERO}[\text{ SIGMA2}]) \end{array}$$

The specifications of the three alternatives are performed, first without scaling the data, ML 6.2:

$$\begin{array}{l} {\text{U}}_{\text{HST}}={\text{ASC}}_{\text{HST}}+{\text{BETA}1}^{*}{\text{P}}_{\text{HST}}+{\text{BETA}2}^{*}{\text{T}}_{\text{HST}}\\ {\text{U}}_{\text{BUS}}={\text{ASC}}_{\text{BUS}}+{\text{BETA}1}^{*}{\text{P}}_{\text{BUS}}+{\text{BETA}2}^{*}{\text{T}}_{\text{BUS}})\\ \;+\text{ZERO}[\text{ SIGMA}]\\ {\text{U}}_{\text{CAR}}={\text{ ASC}}_{CAR}+{\text{BETA}1}^{*}{\text{PT}}_{\text{CAR}}+{\text{BETA}2}^{*}{\text{T}}_{\text{CAR}}\\ \;+\text{ZERO}[\text{ SIGMA}] \end{array}$$

And with the Base 2 data scaled, with the scale factor obtained in the MNL3.1 estimate (μ = 0.0830), the model is specified with three alternatives such that ML.6.4:

$$\begin{array}{l} {\text{U}}_{\text{HST}}={\text{ ASC}}_{\text{HSTE}}+{\text{BETA}1}^{*}{\text{P}}_{\text{HSTE}}+{\text{BETA}2}^{*}{\text{T}}_{\text{HSTE}}\\ {\text{U}}_{\text{BUS}}={\text{ASC}}_{\text{BUS}}+{\text{BETA}1}^{*}{\text{P}}_{\text{BUS}}+{\text{BETA}2}^{*}{\text{T}}_{\text{BUS}})\\ \;+\text{ZERO}[\text{ SIGMA}]\\ {\text{U}}_{\text{CAR}}={\text{ ASC}}_{CARE}+{\text{BETA}1}^{*}{\text{PT}}_{\text{CARE}}+{\text{BETA}2}^{*}{\text{T}}_{\text{CARE}}\\ \;+\text{ZERO}[\text{ SIGMA}] \end{array}$$

Tables 5, 6 show the results for the Logit Mixto models, with three and four alternatives as well (Bierlaire, 2003).

**Table 5:** Results of ML models estimated with four alternatives, with panel effect.

**Models**	**ML5**	**ML5.1**			**ML5.2**		
		**Value**	***T-*****test**		**Value**	***T-*****test**	
Constant Alt. HST1	ASC1	–	–	–	–	–	–
Constant Alt. BUS	ASC2	−0.001	−0.01		1.25	−5.08	^***^
Constant Alt. HST2	ASC3	–	–	–	–	–	–
Constant Alt. CAR	ASC4	−4,2	−14.27	^***^	−0.128	−1.19	
Total travel cost	θ_P_	0,007	2.68	^***^	0	−0.65	
Travel time	θ_T_	−0,028	−8.27	^***^	−0,003	−1.19	
Theta	μ	8,43	1.19		123	1.19	
Sigma1		2,89	15.39	^***^	3.85	13.74	^***^
Sigma2		–	–	–	0.062	0.052	
ZEROSigma1		8,35	7,7	^***^	14.8	6.87	^***^
ZEROSigma2		–	–	–	0.004	0.59	
R-Squared	*R*^2^	0.56			0.574		
Adjusted R-Squared	${\bar{R}}^{2}$	0.559			0.573		
Log-likelihood	I(θ)	−1843,563			−1783,333		
Sample	N	6045			6045		
Generation error distribution	DRAW	Halton	125		Halton	20	
Willingness to pay	VOT	−3,929			24,309		

Value is the estimated coefficient for each of the explanatory variables included in the model. T-test: ^*^ 90%, ^**^ 95%, and

*** *99% level of significance. It is the statistic that allows to contrast the null hypothesis of individual non-significance of each of the variables included in the model, (the contrast is performed through the level of significance associated with that statistic, so that if the level of significance is greater than 0.05 the variable is not statistically significant)*.

**Table 6:** Results of ML models estimated with three alternatives, with panel effect and with scaled data.

**Models**	**ML6**	**ML6.1**			**ML6.2**			**ML6.3**		
		**Value**	***T-*****test**		**Value**	***T-*****test**		**Value**	***T-*****test**	
Constant Alt. HST	ASC1	6.68	6.48	^***^	–	–	–	–	–	–
Constant Alt. BUS	ASC2	6.51	6.42	^***^	−0.669	−1.9	^**^	−0.425	−1.22	
Constant Alt. CAR	ASC3	–	–	–	−51,6	−11.8	^***^	−52.7	−13.12	^***^
Total travel cost	θ_P_	−0.009	−1.09		−0.2	−8.71	^***^	−0.199	−8.66	^***^
Travel time	θ_T_	−0.011	−2.27	^***^	−0.087	−7,84	^***^	−0.087	−78	^***^
Sigma1		5.8	9.21	^***^	4.32	17,41	^***^	4.25	16.51	^***^
ZEROSigma1		33.6	4.61	^***^	18.6	8,71	^***^	18.6	8.25	^***^
R-Squared	*R*^2^	0.391			0.556			0.556		
Adjusted R-Squared	${\bar{R}}^{2}$	0.39			0.554			0.555		
Log-likelihood	I(θ)	−2552,333			−1862,371			−1858,487		
Sample	N	6045			6045			6045		
Generation error distribution	DRAW	Halton	20		Halton	20		Halton	125	
Willingness to pay	VOT	1.187			0.435			0.435		

Value is the estimated coefficient for each of the explanatory variables included in the model. T-test: ^*^ 90%,

** 95%, and

*** *99% level of significance. It is the statistic that allows to contrast the null hypothesis of individual non-significance of each of the variables included in the model, (the contrast is performed through the level of significance associated with that statistic, so that if the level of significance is greater than 0.05 the variable is not statistically significant)*.

The results of the Multinomial Logit models with three choice alternatives (HST-CAR and BUS) improve in terms of the coefficient of determination, showing more realistic payment arrangements. Even in the case of when we include the comfort variable (measured according to the number of transfers), together with the total price of the trip (which separately includes the cost of the trip and the cost of parking for the car, is significant. As well as that obtained from models whose variables are not significant and with signs of time not expected, the results improve in a substantial way.

## Conclusions and recommendations

This article shows an application of the demand models or discreet choice of Logit type to obtain the provisions for the payment of the consumers of a transport service, specifically in the corridor Toledo-Madrid/Madrid Airport. In this case it is analyzed what will be the choice of the travelers that make this journey, considering that they have three alternatives at present, the high speed train, car and the bus.

The theoretical basis of these emodels starts from the theory of random utility specifically in the theory of the maximization of utility. This model is based on the existence of a rational consumer that represents the average behavior of the set of consumers, based on a series of parameters that measure that utility. In the case of transport, these variables are usually the travel time, the cost or price of the service and the convenience of the trip, as well as different socioeconomic variables of the individual.

The model data are obtained through a survey of declared preferences. In it an exercise of simulation of alternative scenarios is proposed that configure mobility in the future. This mobility is based on the new infrastructure of connection with the airport. So there are different service options on the route which are two transfers, one transfer or no transfer.

In general, of the results presented above, we highlight the variables of the equation and the measures of goodness of the model.

Logit models have been estimated that measure the probability of choosing the HST in front of the bus or the car independently and according to the socioeconomic characteristics of the subject and the attributes of the different modes of transport. The variables included in each of the models are: time, price, sex and income. Thus, the attributes for each of the transport modes are (1) HST, price and travel time, (2) bus, price and travel time and (3) car taking into account price, parking cost and travel time.

Specifically, the results show that the willingness to pay HST-Car binomial models is too large (600 € per hour saved on the trip), motivated by individuals who have responded in a lexicographic way and motivated by the smaller number of people who have chosen option bus in front of the HST option. The provisions for the payment of the binomial HST-Bus, are more reasonable and oscillate between 23 and 25 € per hour.

In multinomial models the provisions for payment are more realistic, except when we include the comfort variable, together with the total price of the trip, with which models are obtained whose variables are not significant and even with the signs of the time not expected, estimating The model without independent terms with which you get a result of 26.3 € per hour.

From the results of the modeling we can analyze how the consumer is willing to pay 26.3 € for each hour of travel saved which gives us information about the importance of travel time over the price paid.

In conclusion, based on this information, a sustainable transport policy focusing on HST-airplane intermodal transport is recommended in order to facilitate the reduction of travel times, by encouraging the use of efficient public transport as the high-speed train to reach the airport.

A transport policy focused on replacing private transport with public transport (HST), benefits, on the one hand, users who will have shorter travel times and in general, the transport system to reduce the congestion of access to Madrid and to the Madrid Airport (Li and Hensher, 2012).

The main variables to encourage the integration of air and high-speed modes of transport are the visibility of the offer, integrated management of the reservation and boarding passes, check-in and baggage control, full travel responsibility, the management of passenger loyalty programs, ease of connection in intermodal mode, travel time and price (Muro, 2012). As for this offer, and above all regarding the connections between Toledo and Madrid airport, a new line of direct buses to the airport has emerged (Muro and Pérez, 2016) and recently, in February 2017, the offer of combined train and flying tickets (Train and Fly)^6^ from the city of Toledo, which indicates improvements in air-rail intermodality (*air-rail intermodality*).

## Author contributions

AM: modeling and coordination. IP: methodological framework. SG: introduction and conceptual framework.

### Conflict of interest statement

The authors declare that the research was conducted in the absence of any commercial or financial relationships that could be construed as a potential conflict of interest.

## Footnotes

^1^Based on the neoclassical theory of consumer behavior.

^2^From this set of decision we derive discrete choice models, which we will see below.

^3^This is possible under the assumption of rational consumer behavior, namely perfect rationality (Simon, 1957).

^4^BIOGEME. http://biogeme.epfl.ch/

^5^Assuming that the errors distribute as Gumbel with zero mean and different variance.

^6^http://www.renfe.com/viajeros/trenmasavion/index_ES.html
